# Systematic Analysis of Long Noncoding RNA and mRNA in Granulosa Cells during the Hen Ovulatory Cycle

**DOI:** 10.3390/ani11061533

**Published:** 2021-05-25

**Authors:** Liang Li, Xun Deng, Silu Hu, Zhifu Cui, Zifan Ning, Taotao Gui, Xiaoling Zhao, Diyan Li, Yan Wang, Huadong Yin, Lin Ye, Yaofu Tian, Yao Zhang, Hua Li, Qing Zhu

**Affiliations:** 1 Farm Animal Genetic Resources Exploration and Innovation Key Laboratory of Sichuan Province, College of Animal Science and Technology, Sichuan Agricultural University, Chengdu 611130, China; liliang@stu.sicau.edu.cn (L.L.); dengxun082439@163.com (X.D.); erichu121@foxmail.com (S.H.); 2018102013@stu.sicau.edu.cn (Z.C.); ningzifan@163.com (Z.N.); guitaotao1993@163.com (T.G.); zhaoxiaoling@sicau.edu.cn (X.Z.); diyanli@sicau.edu.cn (D.L.); as519723614@163.com (Y.W.); yinhuadong@sicau.edu.cn (H.Y.); yelin8497@163.com (L.Y.); maikurakitt@163.com (Y.T.); zhangyao12316@163.com (Y.Z.); 2 Guangdong Provincial Key Laboratory of Animal Molecular Design and Precise Breeding, Foshan University, Foshan 528231, China

**Keywords:** chicken, granulosa cell, ovulation, luteinizing hormone surge, lncRNA

## Abstract

**Simple Summary:**

Chicken is one of the most economically important farm poultry, and providing many food products, such as meat and eggs for human consumption. However, follicle transcriptome studies in chickens with timepoints relating to changes in luteinizing hormone level remain unknown. In this study, the largest preovulatory follicle of chicken underwent the early, middle, and terminal stages of ovulatory cycle. Our work provides a comprehensive analysis of lncRNAs and mRNAs in chicken granulosa cells during the ovulatory cycle. A total of 12,479 mRNAs and 7528 lncRNAs were identified among the three stages. Thousands of lncRNAs were annotated, and the most differentially abundant genes were detected in the luteinizing hormone surge stage. Functional features of the lncRNAs and mRNAs at each stage were revealed, which was also associated with the changes in serum luteinizing hormone level. Especially, genes related to oxidative stress, steroids regulation, and inflammatory process were enriched in the luteinizing hormone surge stage, The comprehensive data generated in this study provides the foundation for future investigations to improve the reproductive performance of chickens and explore the mechanisms responsible for female ovarian diseases.

**Abstract:**

Long non-coding RNAs (lncRNAs) and mRNAs are temporally expressed during chicken follicle development. However, follicle transcriptome studies in chickens with timepoints relating to changes in luteinizing hormone (LH) levels are rare. In this study, gene expression in Rohman layers was investigated at three distinct stages of the ovulatory cycle: zeitgeber time 0 (ZT0, 9:00 a.m.), zeitgeber time 12 (ZT12, 9:00 p.m.), and zeitgeber time 20 (ZT20, 5:00 a.m.) representing the early, middle, and LH surge stages, respectively, of the ovulatory cycle. Gene expression profiles were explored during follicle development at ZT0, ZT12, and ZT20 using Ribo-Zero RNA sequencing. The three stages were separated into two major stages, including the pre-LH surge and the LH surge stages. A total of 12,479 mRNAs and 7528 lncRNAs were identified among the three stages, and 4531, 523 differentially expressed genes (DEGs) and 2367, 211 differentially expressed lncRNAs (DELs) were identified in the ZT20 vs. ZT12, and ZT12 vs. ZT0, comparisons. Functional enrichment analysis revealed that genes involved in cell proliferation and metabolism processes (lipid-related) were mainly enriched in the ZT0 and ZT12 stages, respectively, and genes related to oxidative stress, steroids regulation, and inflammatory process were enriched in the ZT20 stage. These findings provide the basis for further investigation of the specific genetic and molecular functions of follicle development in chickens.

## 1. Introduction

Chicken (*Gallus gallus*) is one of the most economically crucial domestic poultry worldwide, providing food products such as meat and eggs for human consumption. In recent decades, the laying performance of layers has been significantly improved by artificial selection [1], coupled with consumer preference for better quality eggs. The ovary is a critical organ in the female reproductive system, directly mediating follicle maturation and secretion of endocrine hormones during the ovulatory cycle, and resulting in fecundity in chickens [2,3]. In laying chickens, a strict follicular hierarchy is maintained, which comprises the large yellow follicle (usually F1–F5), small yellow follicles (SYF), and small white follicles (SWF) [4]. The dominant follicle rapidly develops from the F5 follicle to the F1 follicle before ovulation. Subsequently, the mature F1 follicle is triggered to ovulate from the ovary by the daily luteinizing hormone (LH) surge [5,6]. The stability of the ovulatory cycle is vital for high laying performance in chickens. The ovulatory cycle usually lasts approximately 24–25 h [7] and is reported to be synchronized by a surge of LH secretion [8,9]. Granulosa cells also play an essential role in follicle development and ovulation [10,11] by synthesizing estradiol and progesterone in response to the stimulation of LH [12], and this synthesis process predominantly occurs in the largest yellow follicle (F1) [13].

Several studies have analyzed the chicken transcriptome during follicle development at different hierarchy follicles, including follicle selection and growth [14,15,16,17]. For example, Shen et al. performed a comprehensive chicken circRNAome during three development stages of follicular theca cells, including small yellow follicles (SYF), the smallest hierarchical follicles (F6), and the largest hierarchical follicles (F1). A total of 5622 circRNAs were identified in all developmental stages, which involved metabolic and immune processes [18]. Several studies evidence that lncRNAs are instrumental in follicular development [19,20,21]. However, chicken transcriptome studies based on LH level changes have not yet been performed during the ovulatory cycle.

In this study, a comprehensive analysis of granulosa cells of the largest hierarchical follicles (F1) in chickens at zeitgeber time 0 (ZT0), zeitgeber time 12 (ZT12), and zeitgeber time 20 (ZT20) stages, which are closely linked to the regulation of the hypothalamus-pituitary-ovary axis to maintain the ovulatory cycle, was performed. Ribo-Zero RNA sequencing (RNA-seq) analysis of the granulosa cells revealed the enriched functional features at each stage. The findings of this study will facilitate a better understanding of transcriptomic changes in chicken follicle development, especially those obtained based on changes in serum LH levels, and will also provide a reference for future studies on female ovarian diseases.

## 2. Materials and Methods

### 2.1. Animal Preparation and Sample Collection

Three hundred healthy Rohman layers were housed with water ad libitum and exposed to a 16-h light and 8-h dark photoperiod per day. The oviposition time of each chicken was monitored from 6:00 a.m. to 6:00 p.m. for 70 days and was precisely recorded to predict the ovulatory cycle. At 210 days old, chickens that stably lay eggs between 7:00 and 8:00 a.m. were selected for further experiments. LH concentration was measured in chicken serum during the ovulatory cycle (4-h intervals from ZT0 to ZT24 and six repetitions for each stage) using LH ELISA Test Kits (Shanghai Jianglai Biotech, Shanghai, China) according to the manufacturer’s instructions. Chickens were randomly assigned into three groups (*n* = 3 for each group), which were euthanized at 9:00 a.m. (ZT0), 9:00 p.m. (ZT12), and 5:00 a.m. (ZT20), respectively. Granulosa cells were harvested from the largest preovulatory follicle (F1), immediately transferred into liquid nitrogen, and then stored at −80 °C until subsequent RNA isolation. The F1 follicle in each stage was also embedded in paraffin and stained with hematoxylin and eosin (H&E) to evaluate follicle growth.

### 2.2. RNA Extraction, Library Construction, and Sequencing

Total RNA was extracted using TRIzol reagent (Invitrogen, CA, USA) following the manufacturer’s instructions. The integrity and concentration of total RNA were evaluated using an Agilent 2100 Bioanalyzer (Agilent Technologies, Palo Alto, CA, USA) and NanoDrop spectrophotometer (Nanodrop Technologies, Wilmington, DE, USA). RNA-seq complementary DNA (cDNA) libraries were generated with the Ribo-ZeroTM Gold Kit (Illumina, San Diego, CA, USA) and then sequenced on an Illumina NovaSeq platform at Novogene Corporation (Beijing, China). Further analyses were based on high-quality data.

### 2.3. Transcriptome Annotation and Classification

Processed reads from each sample were mapped to the chicken reference genome (Gallus_gallus v.6.0 from Ensemble) using STAR (v2.6.0c) [22] to identify mRNA transcripts. LncRNA transcripts were then obtained with the following protocol: (1) mapped reads of the samples were assembled by Cufflinks (v.2.1.1) [23] and transcripts with a clipped exon (first or last exon with length no more than 15 nt) and expression levels lower than 0.1 were removed using a custom script (Additional file 1); (2) Transcripts (transcript length ≤ 250 bp) were removed using AssemblyLine (v.0.2), and remaining transcripts were merged using TACO (with the primary function: taco run) [24]; (3) Transcripts that were annotated as protein-coding genes (PCG) were filtered out with a custom script (Additional file 1), and the remaining transcripts were performed to exclude any protein-coding potential; (4) To identify the coding potential of lncRNAs, the putative non-coding transcripts were translated into their possible protein sequences (all six reading frames) with Transeq, which is part of the EMBOSS package. Significant domain hits against the Pfam (v.31) database were calculated using PfamScan (v.1.6) [25] and PCG transcripts in the reference genome. CPC2 (coding potential calculator v.2.0) [26] was used to assess coding potential in both strands of the remaining sequences, and the transcripts labeled as “non-coding” in the output result were retained. Transcripts identified as non-coding by CPC2 and PfamScan were considered to be lncRNAs. Options for the various bioinformatics tools were also presented in Additional File 1. To classify lncRNAs, the classifier module of the program FEELnc (v.0.2) [27] was utilized, which can categorize lncRNAs based on their genomic localization.

### 2.4. Differential Expression Analysis

Quantification of lncRNA and mRNA expression in each sample was calculated by Kallisto (v2.1.1) [28]. The mRNA and lncRNA that an expression value greater than 0.1 transcripts per kilobase million (TPM) in at least one library were considered expressed and were selected for further differential expression analysis. The differential expression analysis was performed using the DESeq2 R package (v3.12), with |log2 (fold change)| > 1 and adjusted *p*-value < 0.05 as the cut-offs for statistical significance.

### 2.5. Short Time-Series Expression Miner Analysis

Differentially expressed lncRNAs (DELs) and differentially expressed genes (DEGs) were clustered using the short time-series expression miner (STEM) clustering algorithm to identify temporal gene expression profiles in chicken granulosa cells during the ovulatory cycle. The colored modules profiles were significantly enriched clusters (Bonferroni-adjusted *p*-value < 0.05).

### 2.6. Functional Enrichment Analysis

The functions of lncRNAs were predicted based on the cis targets that were co-localized with DEGs. DEGs within 100 kb upstream and downstream of the lncRNA were considered as nearby protein-coding genes. Pearson’s correlation coefficients between the expression of DELs and DEGs were calculated using Hmisc (an R package from https://cran.r-project.org/, accessed on 7 May 2021 v4.5.0), and the cut-offs |r| > 0.95 and *p*-value < 0.05 were considered to indicate a positive correlation between DELs and DEGs. The DEGs and cis targets were subjected to Gene Ontology (GO) enrichment analysis and Kyoto Encyclopedia of Genes and Genomes (KEGG) functional enrichment analysis using the Metascape online tool [29]. GO terms and KEGG pathways with a Benjamini-adjusted *p*-value < 0.05 were considered significantly enriched in each list of up- and down-regulated DEGs and DELs.

### 2.7. Cell Culture and LH Treatment

Granulosa cells (from the chicken follicle) were cultured in Dulbecco’s modified Eagle’s medium (DMEM, Gibco, Carlsbad, CA, USA) containing 10% FBS (Gibco) and 1% penicillin/streptomycin (Gibco) at 37 °C in a humidified 5% CO_2_ atmosphere. Cells were reset with 100 nM dexamethasone (DXM, Sigma Chemical Co., St. Louis, MO, USA) for 1 h, washed twice with a serum-free medium by centrifugation at 200× *g* for 5 min, and were then treated with 100 ng/mL LH (Sigma Chemical Co.) for 24 h. Cells without LH treatment were used as a control. After incubation for 24 h, the cultured cells were harvested for RNA isolation to validate the lncRNA and mRNA expression.

### 2.8. Quantitative Real-Time PCR Validation

Primers for DELs and DEGs (Table S1) were designed using Primer 3.0 software and checked with the NCBI Primer-BLAST tool. cDNAs were synthesized from RNA using a PrimeScript™RT Kit with gDNA Eraser (Takara Biotechnology Co., Dalian, China). Quantitative real-time PCR analysis was performed with SYBR Premix Ex Taq II kit (Takara Biotechnology Co., China) using a CFX96 Real-Time PCR detection system (Bio-Rad, Richmond, CA, USA). Relative expression values were obtained using the 2^−ΔΔCT^ method and normalized using the housekeeping gene *GAPDH*.

### 2.9. Statistical Analysis

Statistical analysis was performed using SigmaPlot 12.0 and Microsoft Excel. Student’s *t*-test or one-way ANOVA followed by Tukey’s HSD (Honestly Significant Difference) test were used to analyze gene expression in multiple comparisons. Pearson’s correlation analysis was used for pairwise comparisons of RNA-seq and qRT-PCR data.

## 3. Results

### 3.1. Changes in LH level and Histological Analyses of Chicken Follicle

**Figure 1:**
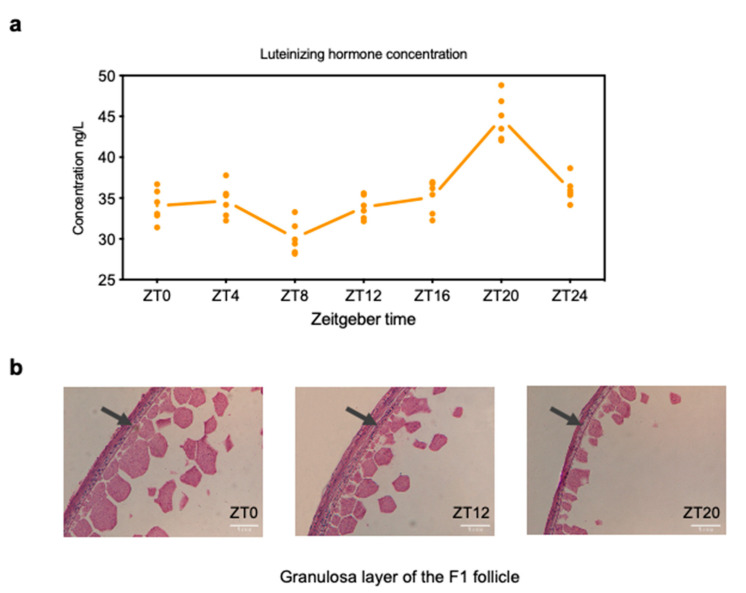
Changes in serum luteinizing hormone (LH) level and H&E-stained follicle granulosa layer among three stages of the ovulatory cycle. (**a**) LH concentration was measured in chicken serum during the ovulatory cycle (4-h intervals from ZT0 to ZT24 and six repetitions for each stage), and the highest LH level was detected at the ZT20 stage. (**b**) Chicken F1 follicles were embedded in paraffin and stained with hematoxylin and eosin (H&E) at ZT0, ZT12, and ZT20. ZT0 (9:00 a.m.), ZT12 (9:00 p.m.), and ZT20 (5:00 a.m.) represent the early, middle, and LH surge stages, respectively, of the ovulatory cycle.

Chicken oviposition was monitored for 70 days to ascertain ovulation timing and obtain samples with the same ovulatory cycle. LH levels in chicken serum were determined and the highest concentration was recorded at the ZT20 stage (44 ng/L) (Figure 1a). Nine chickens that ovulated simultaneously (approximately 8:00 a.m.) and continued to lay for at least 30 days were selected for further experiments. The chickens were randomly assigned to three groups—ZT0 ZT12, and ZT20—representing distinct stages of the ovulatory cycle. H&E staining of the F1 follicle in each group showed that the granulosa layer of the follicle at ZT20 was thinner with fewer granulosa cells than the ZT12 and ZT0 stages (Figure 1b).

### 3.2. Expression Pattern of lncRNAs and mRNAs

A total of 153.64 Gb of raw data with 150-bp paired-end sequences were generated. Low-quality and adaptor sequences were filtered out, and an average of ~16.9 Gb high-quality RNA-seq data remained (Table 1), which were then subjected to biological information analysis.

From the filtered data, 12,479 mRNAs and 7528 candidate lncRNAs (TPM > 0.1 in at least one replicate) were identified. Expression of lncRNAs was lower than that of the mRNA transcripts, and the transcript length of lncRNAs was also shorter than that of the mRNA transcripts (Additional file 2a,b). The lncRNAs had fewer exons compared with the mRNAs; the average number of exons in the lncRNAs was two, while that in the mRNAs was seven (Additional file 2c, Table S2), and the sequences of lncRNAs were available in Additional file 3. The lncRNAs were classified into eight groups, including 728 antisense exon lncRNAs, 369 antisense intron lncRNAs, 678 intergenic lncRNAs, 1920 intronic lncRNAs, 292 sense same strand lncRNAs, 126 convergent lncRNAs, 205 divergent lncRNAs, and 3210 other lncRNAs (Additional file 1d).

**Figure 2:**
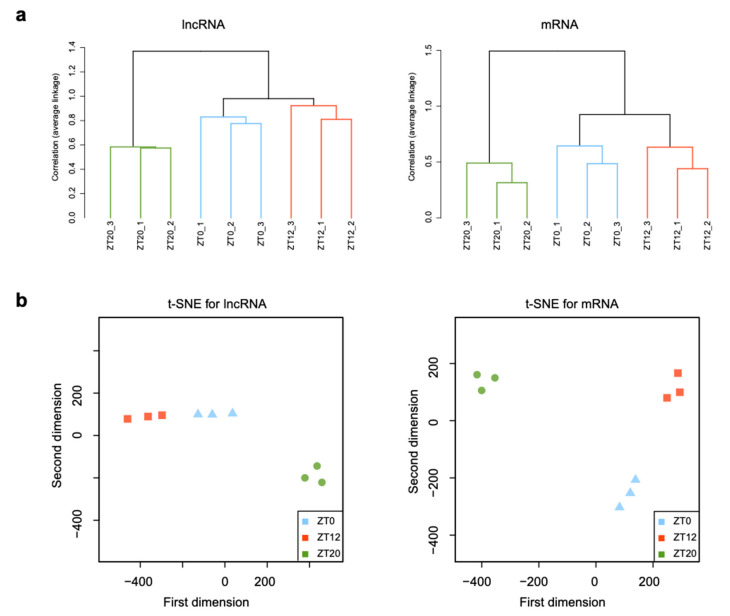
Global gene expression profiles of the F1 follicle during development. Hierarchical clustering of all samples (**a**) and t-SNE plot (**b**) of lncRNAs and mRNAs based on expression profiles in different development stages. The top and left panel is the sample and gene tree, and the value represents the log2 transformed values of (TPM + 1). ZT0 (9:00 a.m.), ZT12 (9:00 p.m.), and ZT20 (5:00 a.m.) represent the early, middle, and LH surge stages, respectively, of the ovulatory cycle.

To reveal dynamic expression changes in RNA transcripts from different stages of the F1 follicle during the ovulatory cycle, hierarchical clustering analyses were performed on transcripts among three different developmental stages, showing maximal expression in granulosa cells of the F1 during the ovulatory cycle (Figure 2a). The t-distributed stochastic neighbor embedding (t-SNE) analysis was performed based on lncRNA and mRNA expression profiles (Figure 2b). The findings revealed that the samples were grouped into two distinct groups based on the developmental stage: the pre-LH surge stage (ZT0 and ZT12 stages) and the LH surge stage (ZT20 stage). Thus, expression patterns of the same gene in each stage of the ovulatory cycle were consistent between samples, and all samples from the three stages typically clustered into distinct groups. A greater difference was detected both in lncRNAs and mRNAs expression between the ZT12 and ZT20 stages, while a similar expression was observed between the ZT0 and ZT12 stages. These findings indicated that gene expression levels were correlated among the biological replicates of each stage, and genes had a similar expression in the pre-LH surge stage, but a distinct expression occurred in the LH surge stage. The comprehensive catalog of lncRNAs and mRNAs in the chicken follicle generated in this study could serve as a foundation for understanding the functions of lncRNAs and mRNAs in chicken follicle development during the ovulatory cycle.

### 3.3. Functional Enrichment Analysis of Differentially Expressed mRNAs

**Figure 3:**
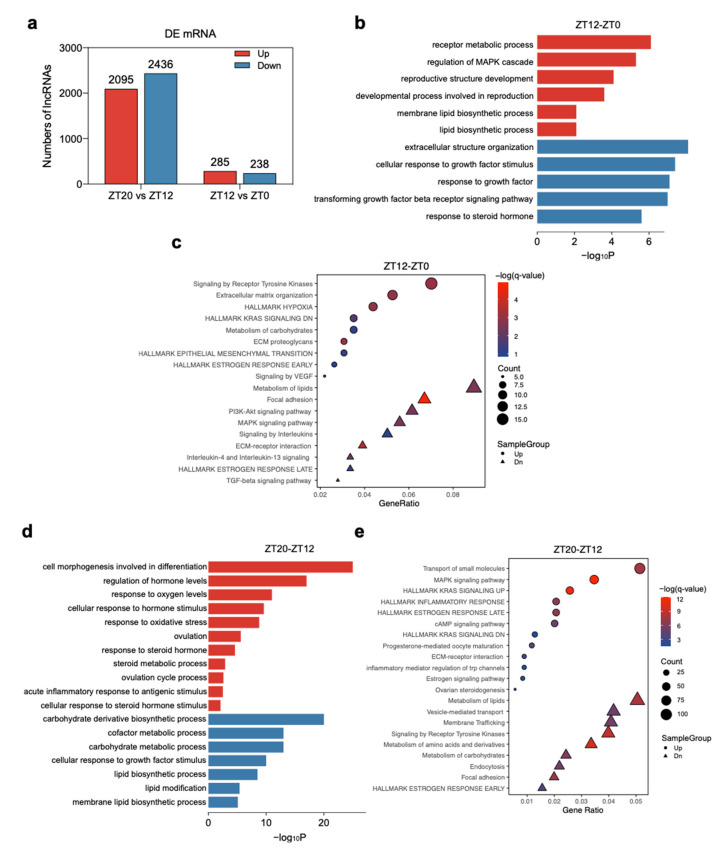
Gene ontology (GO) and pathway analysis of differentially expressed genes (DEGs) during follicle development in the chicken ovulatory cycle. (**a**) Number of DEGs in pairwise stages. (**b**) Enriched GO terms (top) and (**c**) pathways (bottom) of DEGs in ZT12 vs. ZT0 group. (**d**) Enriched GO terms (left) and (**e**) pathways (right) of DEGs in ZT12 vs. ZT0 group. Directly up-regulated (red) and down-regulated (blue) gene numbers and ontologies are shown.

Changes in plasma LH levels indicated that LH concentration reached a relatively high level in the ZT20 stage (44 ng/L) during the ovulatory cycle (Figure 1a). Therefore, functional enrichment analysis of the DEGs between the ZT12 vs. ZT0 group and ZT20 vs. ZT12 group was conducted to investigate the physiological changes acting on the F1 follicle during the ovulatory cycle. A total of 523 and 4531 DEGs were detected in the ZT12-ZT0 and ZT20-ZT12 groups, respectively. Of these DEGs, 285 were up-regulated and 238 were down-regulated in the ZT12-ZT0 group, while 2095 were up-regulated and 2436 were down-regulated in the ZT20-ZT12 group (Figure 3a, Table S3). These results indicated a distinct expression pattern occurred between the ZT20 and ZT12 stages. Besides, more DEGs were detected in the ZT20-ZT12 group than in the ZT12-ZT0 group. These findings were also consistent with the results of hierarchical clustering and t-SNE analyses, suggesting that a massive physiological change related to the surge of LH concentration occurred at the ovulatory period of follicle development.

Functional enrichment analyses of the DEGs in the ZT12-ZT0 and ZT20-ZT12 groups revealed that the DEGs up-regulated in the ZT12-ZT0 group were enriched in receptor metabolic process (GO:0043112), membrane lipid biosynthetic process (GO:0046467), and lipid biosynthetic process (GO:0008610), hallmark estrogen response early (M5906), response to progesterone (GO:0032570) (Figure 3b,c, Table S4), while the DEGs down-regulated in the ZT12-ZT0 group were mainly enriched in the cellular response to growth factor stimulus (GO:0071363), transforming growth factor-beta receptor signaling pathway (GO:0007179), response to steroid hormone (GO:0048545), MAPK signaling pathway (hsa04010), and response to steroid hormone (GO:0048545) (Figure 3b,c, Table S4). Similarly, DEGs up-regulated in the ZT20-ZT12 group were enriched in cell morphogenesis involved in differentiation (GO:0000904), regulation of hormone levels (GO:0010817), response to oxygen levels (GO:0070482), cellular response to hormone stimulus (GO:0032870), steroid metabolic process (GO:0008202), and response to steroid hormone (GO:0048545). The up-regulated DEGs in this group were also enriched in regulation of inflammatory response (GO:0050727), acute inflammatory response to antigenic stimulus (GO:0002438), ovulation (GO:0030728), ovulation cycle process (GO:0022602), progesterone-mediated oocyte maturation (hsa04914), and estrogen signaling pathway (ko04915) (Figure 3d,e, Table S4). DEGs down-regulated in the ZT20-ZT12 group were mainly enriched in lipid biosynthetic process (GO:0008610), lipid modification (GO:0030258), cellular response to lipid (GO:0071396), and metabolism of lipids (R-HSA-556833). (Figure 3d,e, Table S4).

### 3.4. Functional Enrichment Analysis of Differentially Expressed lncRNAs

**Figure 4:**
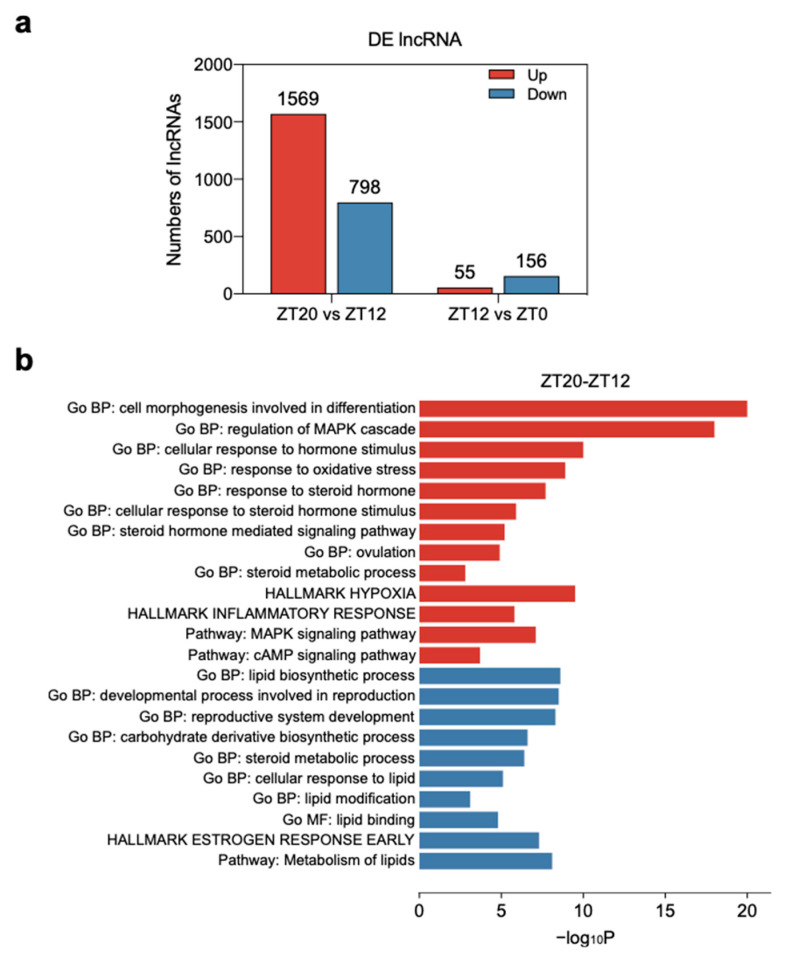
Gene ontology (GO) and pathway analysis of differentially expressed genes (DEGs) during follicle development in the chicken ovulatory cycle. (**a**) Number of DELs in pairwise stages. (**b**) Enriched GO terms (up) and pathways (bottom) of DEGs in ZT20 vs. ZT12 group. The up-regulated (red) and down-regulated (blue) gene numbers and gene ontology terms are shown.

A total of 211 and 2367 DELs were detected in the ZT12-ZT0 and ZT20-ZT12 groups, respectively. Of these DELs, 55 were up-regulated and 156 were down-regulated in the ZT12-ZT0 group, while 1569 were up-regulated and 798 were down-regulated in the ZT20-ZT12 group (Figure 4a, Table S5). Consistent with the DEGs data, more DELs were detected in the ZT20-ZT12 group than in the ZT12-ZT0 group. LncRNAs can act alongside cis factors, but diffusion or transport to other cellular compartments render these lncRNA transcripts too dilute to mediate a plausible function [30]. Therefore to predict the functional role of lncRNAs identified in this study, the DEGs within the 100-kb upstream and downstream regions of each DEL were assumed to act as cis target genes [31]. Only a few cis target genes of DELs in the ZT12-ZT0 group were detected, therefore functional enrichment analysis focused on DEGs located near the DELs in the ZT20-ZT12 group to explore their functions during the LH surge period. Targets of up-regulated DELs in the ZT20-ZT12 group were related to cell morphogenesis involved in differentiation (GO:0000904), cellular response to hormone stimulus (GO:0032870), signaling by receptor tyrosine kinases (R-HSA-9006934), regulation of MAPK cascade (GO:0043408), response to steroid hormone (GO:0048545), and response to oxygen levels (GO:0070482) (Figure 4b, Table S6). On the other hand, targets of down-regulated DELs in the ZT20-ZT12 group were enriched in lipid biosynthetic process (GO:0008610), the developmental process involved in reproduction (GO:0003006), hallmark estrogen response early (M5906), cellular response to lipid (GO:0071396), and metabolism of lipids (R-HSA-556833) (Figure 4b, Table S6).

### 3.5. Temporal Gene Expression Patterns of mRNAs and lncRNAs

**Figure 5:**
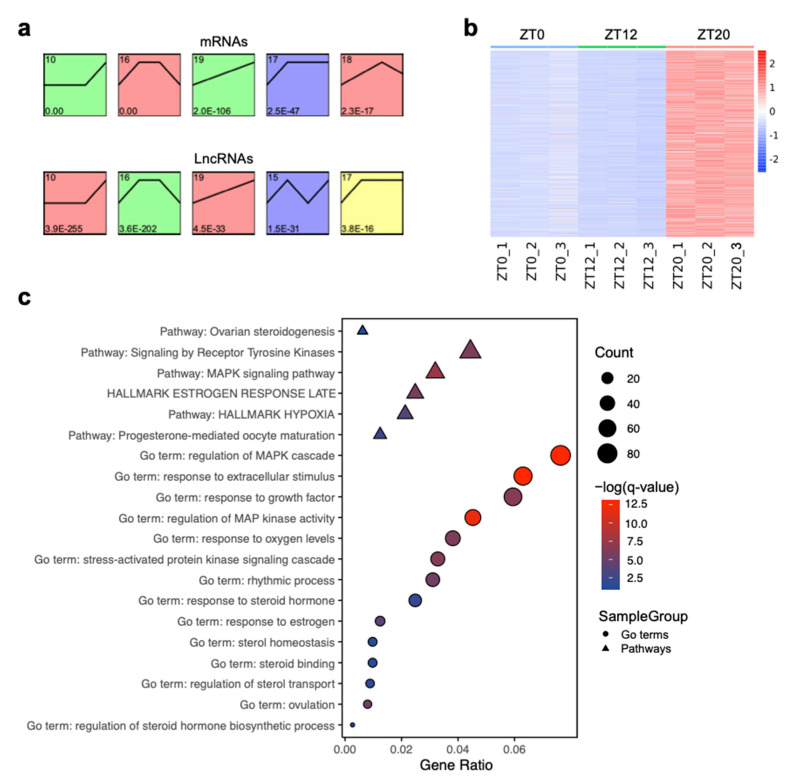
Expression patterns of the time-series clusters. (**a**) STEM analysis identified the temporal expression profiles of differentially expressed genes (DEGs) and differentially expressed lncRNAs (DELs) with *p* < 0.05. The profile number in the top left corner of each profile box was assigned by STEM. The number in the bottom left represents the adjusted *p*-value. (**b**) A heatmap of the transcripts’ expression in cluster 10 based on normalized data of expression values. (**c**) Enriched GO terms (circle) and pathways (triangle) of DEGs in the cluster 10 module.

Global expression patterns of the lncRNAs and mRNAs were further examined by performing a time-series analysis using the STEM tool to identify clusters of DELs and DEGs with similar patterns of expression across three developmental stages of the ovulatory cycle. A total of 3829 mRNAs and 1878 lncRNAs were assigned into nine clusters, respectively (Additional file 4), of which three and four clusters were significantly enriched (*p*-value ≤ 0.05) for mRNAs and lncRNAs, respectively (Figure 5a, Tables S7 and S8). In these cluster profiles, the expression patterns of mRNAs and lncRNAs were similar during the ovulatory cycle. For example, cluster 10 featured in the profiles of both mRNAs and lncRNAs, with 1442 mRNAs and 894 lncRNAs assigned to this cluster, and showing a significant difference in gene expression between the ZT20 and ZT12 stages (Figure 5b). These clusters with a similar expression pattern for mRNAs and lncRNAs suggested that the functions of lncRNAs may be predominantly correlated with these mRNAs. Therefore, the role of mRNAs in cluster 10 was analyzed. The genes in cluster 10 were primarily enriched in response to extracellular stimulus (GO:0009991), regulation of MAPK cascade (GO:0043408), hallmark estrogen response early (M5906), response to oxygen levels (GO:0070482), response to steroid hormone (GO:0048545), sterol homeostasis (GO:0055092), hallmark inflammatory response (M5932), and ovulation (GO:0030728) (Figure 5c, Table S9). As these cluster 10 genes were highly expressed at the ZT20 stage, the enriched functions were congruent with previous results. Thus, these findings suggested that the dynamic function of the F1 follicle was correlated to changes in LH level and consistent with the results mentioned above during the ovulatory period.

### 3.6. Dynamic Expression of Follicular Steroid-Related Genes

**Figure 6:**
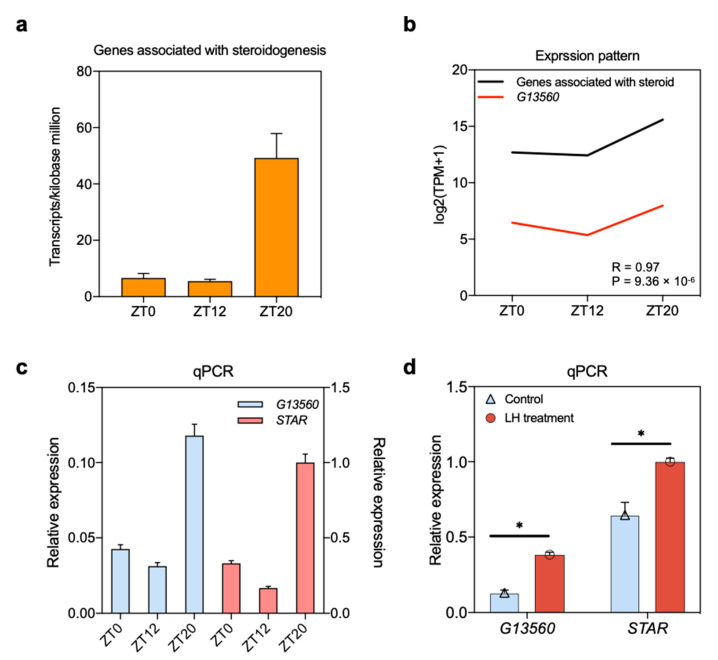
Expression and function of lncRNA *G13560* in follicle development during the chicken ovulatory cycle. (**a**) Expression of genes associated with steroid regulation was up-regulated in the ZT20 stage compared with the other two stages. (**b**) lncRNA was co-expressed with these steroid-related genes and displayed a similar expression pattern. (**c**) lncRNA *G13560* expression was confirmed by qRT-PCR among the three development stages. Data are shown as mean ± SD. (**d**) Co-expression of lncRNA and mRNA was validated in cultured granulosa cells with or without LH treatment. *STAR*, steroidogenic acute regulatory protein; *GAPDH* was used as a control. Data are means ± SD and are representative of at least three independent experiments. * *p* < 0.05. ZT0 (9:00 a.m.), ZT12 (9:00 p.m.), and ZT20 (5:00 a.m.) represent the early, middle, and LH surge stages, respectively, of the chicken ovulatory cycle.

To investigate changes in gene expression during follicle development, the dynamic expression of steroid-related genes at the ZT0, ZT12, and ZT20 stages was explored. There were more transcripts per kilobase million (TPM) at the ZT20 stage than at the other two stages (Figure 6a). Due to the lack of lncRNA annotation libraries, gene expression correlation among samples was calculated to predict functional coregulation. A correlation analysis of lncRNAs and DEGs associated with steroid, including 75 genes enriched in the cellular response to steroid hormone stimulus (GO:0071383), response to steroid hormone (GO:0048545), and steroid metabolic process (GO:0008202) was then performed. The lncRNA *G13560* with a high abundance was similar to that of the steroid-related genes (Figure 6b). The lncRNAs were also up-regulated at the ZT20 stage, and their expression patterns in each stage were validated using qPCR (Figure 6c). Further validation was achieved by co-expressing both lncRNA with mRNA in cultured granulosa cells and treating with or without LH, which was to identify the impact of LH on the expression of transcripts. Expression of both lncRNA and mRNA were up-regulated following the LH treatment (Figure 6d). Based on these results, lncRNA *G13560* may be involved in regulating steroid signaling and associated with changes in LH levels during the ovulatory period.

### 3.7. Validation of mRNAs and lncRNAs

**Figure 7:**
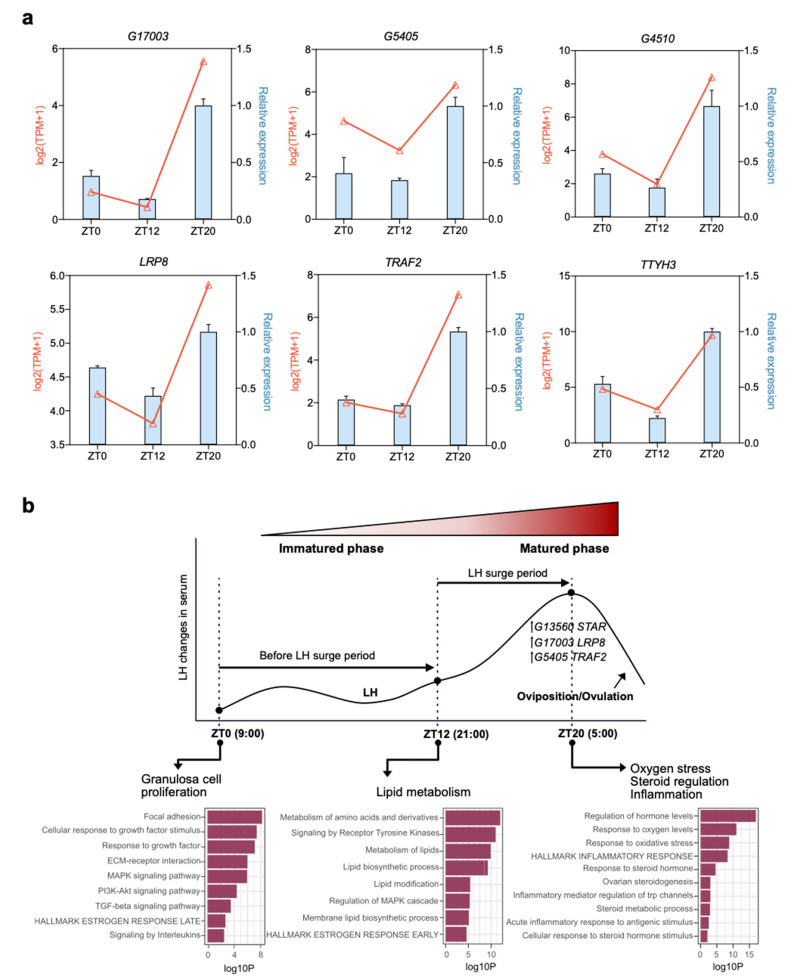
qPCR verification of differentially expressed genes (DEGs) and differentially expressed lncRNAs (DELs) during follicle development. (**a**) Expression patterns of three DELs (*G17003*, *G5405*, and *G4501*) and three DEGs (*LRP8*, *TRAF2*, and *TTYH3*) were validated by qPCR. The *x*-axis represents the three developmental stages during the ovulatory cycle. The *y*-axis represents the relative expression levels of each gene; the left blue color represents relative expression by qPCR, and the right red color is the value of RNA-seq data with log2(TPM + 1). Data are shown as means ± SD and are representative of at least three independent experiments. (**b**) Transcriptome-wide RNA sequencing dynamics to reveal main functional factors of granulosa cell during follicle development. ZT0 (9:00 a.m.), ZT12 (9:00 p.m.), and ZT20 (5:00 a.m.) represent the early, middle, and LH surge stages, respectively, of the chicken ovulatory cycle.

The accuracy of the identified transcriptome expression results in each stage was verified by selecting three paired DELs and DEGs (*G17003*-*LRP8*, *G5405*-*TRAF2*, and *G4510*-*TTYH3*) and validating their expression patterns from RNA-seq results by qPCR (Figure 7a). The relative expression pattern of DELs and DEGs of each stage determined by quantitative real-time PCR was compared with the transformed log2 (TPM + 1) values of RNA-Seq. The DELs and DEGs were expressed in granulosa cells at the three development stages and the expression pattern of DELs with DEGs was consistent with the calculated results from the RNA-seq data, and the high expression levels occurred at the ZT20 stage (Figure 7b). These results illustrated the reliability of our RNA-seq data and the usefulness of the analysis method in the relationship predication of lncRNAs and mRNAs.

## 4. Discussion

Non-coding and protein-coding RNAs have been widely studied in the reproductive system, including in ovary, oocyte, and granulosa cells [32,33,34]. However, studies on chicken follicle development based on changes in LH concentration to explore the functional coregulation of lncRNAs and mRNAs are rare. In this study, a comprehensive analysis of lncRNAs and mRNAs according to the daily serum LH level before ovulation was conducted, and functional features of follicle development both in mRNA and lncRNA levels were identified during the ovulatory cycle (Figure 7b). Furthermore, the expression levels of highly abundant lncRNA and the co-expressed mRNA were verified in cultured granulosa cells, which were treated with LH to mimic a surge of LH in vivo (Figure 6).

Domestic laying hens ovulate approximately every 24 h and thus act as an ideal animal model for investigating the biological process of follicle maturation and ovulation. The F1 follicle, known as the largest yellow follicle before ovulation, is intricately linked to the regulation of the hypothalamus-pituitary-ovary axis to maintain the ovulatory cycle. In this study, the oviposition time was monitored to obtain a chicken population with a regular ovulatory cycle of approximately 24 h. To identify the functional features of lncRNAs and mRNAs in the F1 follicle before ovulation events, three stages of the ovulatory cycle were selected based on the changes in serum LH concentration (Figure 1). Additionally, the current study showed that chicken oviposition occurs before the ZT0 stage, suggesting that the LH surge could trigger ovulation events after the ZT20 stage to maintain the ovulatory cycle.

The F1 follicle could influence follicular development and the ovulatory cycle by regulating hormone levels. Secretion of progesterone is sensitive to the LH surge [35], which occurs in granulosa cells of the F1 follicle. This complicated process of events requires a highly orchestrated program of gene expression and is regulated by various factors. According to the present study, lncRNAs and mRNAs tended to display a stage-dependent expression profile, consistent with other studies [36,37,38]. Hierarchical clustering and t-SNE analyses demonstrated that expression profiles between the ZT20 and ZT12 stages were markedly different from those of the ZT12 and ZT0 stages (Figure 2a,b), suggesting that the expression changes of lncRNAs and mRNAs were associated with serum LH level during the ovulatory cycle. By contrast, the similar expression profiles at the lncRNA and mRNA levels of the ZT0 and ZT12 stages before the LH surge implies that massive molecular and physiological changes associated with follicle development occurred during the LH surge period. Additionally, an identical pattern was observed between lncRNA and mRNA expression, consistent with a study of Yang et al. [39], indicating that the dynamic changes in lncRNA and mRNA transcriptomes function coordinately in related physiological processes. Briefly, these results suggest that the samples were reliable and could be used for further analyses.

In the present study, ZT0, ZT12, and ZT20 represent three different stages of F1 follicle development during the ovulatory cycle. Enrichment analysis of DEGs revealed that almost all stages were enriched in follicle development-related terms (Figure 3). Before the LH surge stage, the enriched GO and pathway terms among the up-regulated DEGs at the ZT0 and ZT12 stages were predominantly related to developmental processes and metabolism processes (Figure 3). The ZT0 and ZT12 stages prior to the LH surge had many overlapping functional enrichment terms, including response to growth factor (GO:0070848), transforming growth factor beta receptor signaling pathway (GO:0007179), TGF-beta signaling pathway (hsa04350), response to growth factor (GO:0070848), and lipid biosynthetic process (GO:0008610) (Figure 3b,c, Table S4). This indicated that granulosa cells of the F1 follicle continued to proliferate to meet the deposition of yolk until the F1 follicle reached matured phase, consistent with the results of other studies [40,41]. The ZT0 and ZT12 stages represent the early and middle stages, respectively, of the ovulatory cycle. Follicle theca cells produce androgens that could diffuse to granulosa cells and then convert to estrogens in response to follicle-stimulating hormone (FSH). FSH is known to stimulate the proliferation of immature granulosa cells via multiple signaling pathways, such as the PI3K/AKT pathway, which was enriched at the ZT0 stage and is involved in cell proliferation [42,43]. The gene *PDGFA*, encoding platelet-derived growth factor subunit A protein, is enriched in the PI3K/AKT pathway and had a higher level of expression at the ZT0 stage, suggesting that granulosa cells were highly proliferative at the early of the ovulatory cycle, consistent with results of other studies [44,45]. Moreover, lipid-related genes were mainly enriched at the ZT12 stage, including lipid biosynthetic process (GO:0008610), membrane lipid biosynthetic process (GO:0046467), lipid transport (GO:0006869), and metabolism of lipids (R-HSA-556833) (Figure 3b,c, Table S4). In the F1 follicle, the deposition of large amounts of yolk lipids was associated with cell proliferation and very-low-density lipoprotein transportation. Genes related to lipid metabolism were enriched at the ZT12 stage, which are essential for promoting the deposition of yolk protein and synthesis of lipids before the LH surge stage [46], For example, *FASN* is involved in lipid metabolism to regulate steroidogenesis in granulosa cells [47], and the observation that this gene was enriched in the ZT12 stage was consistent with the results of another study [48]. These findings suggested that cell proliferation and lipid metabolism in granulosa cells plays a pivotal role in follicle development before the LH surge stage [49,50,51].

In the LH surge stage (ZT20 stage), the up-regulated transcripts were mainly related to oxygen stress, steroid regulation, and ovulation events (Figure 3d,e, Table S4), and these can be used to characterize features of the F1 follicle during the ovulatory period. According to the results of enrichment analysis, four DEGs were related to stimulation (GO:0032870: response to oxygen levels, GO:0000302: response to reactive oxygen species, and GO:0006979: response to oxidative stress), four DEGs related to steroids regulation (GO:0071383: cellular response to steroid hormone stimulus, GO:0048545: response to steroid hormone, and GO:0008202: steroid metabolic process). Genes related to oxidative stress and steroids regulation were highly expressed in the ZT20 stage, which imply that these genes play a crucial role in the regulation of steroids regulation in granulosa cells, and this is in line with the findings that oxidative stress had an impact on the production of steroid hormones in granulosa cell [52]. In addition, the occurrence of oxidative stress was linked to the excessive production of reactive oxygen species, which is a mediator of follicle ovulation [53]. Additionally, target genes of DELs involved in oxidative stress were also enriched at the ZT20 stage (Figure 4b, Table S6). Thus, these findings indicated that oxidative stress and steroid regulation contribute to the ovulatory events [54,55] when the follicle reached the matured phase at the ZT20 stage. Furthermore, up-regulated DEGs at the ZT20 stage (LH surge stage) were also related to inflammatory processes, including acute inflammatory response to antigenic stimulus (GO:0002438), hallmark inflammatory response (M5932), and regulation of inflammatory response (GO:0050727) (Figure 3d,e, Table S4). The ovulatory process is initiated when the LH concentration increases above circulating levels present during the ovulatory cycle [56], therefore the F1 follicle reached the mature phase at the ZT20 stage. Results from the current study showed that many genes with high expression levels at the ZT20 stage are routinely associated with the inflammatory process. This implies that the surge in LH activates specific signaling cascades and transcription factors that initiate the preovulatory follicles to undergo complex processes, and these processes display many similarities to inflammatory processes. These findings in line with many studies showed there is a high degree of analogy between inflammation and ovulation events, and that the ovary follicle undergoes in response to an ovulatory LH stimulus [57,58,59,60,61]. For example, *RGS16* was highly expressed at the ZT20 stage and encodes a regulator of G protein signaling 16, which modulates inflammation [62], is involved in the LH cascade in granulosa cells [63], and is necessary for EGFR-mediated follicle ovulation [5,64,65,66]. Thus, the inflammatory process enriched at the ZT20 stage could contribute to the ovulatory events in the chicken.

Time-series analysis was adopted to explore the dynamic expression pattern of lncRNAs and mRNAs, which revealed that both lncRNAs and mRNAs were dynamically expressed and had similar expression patterns during the ovulatory cycle. DEGs in cluster 10 were up-regulated at the ZT20 stage and were mainly related to oxidative stress, steroids regulation, and ovulation events, including response to growth factor (GO:0070848), stress-activated protein kinase signaling cascade (GO:0031098), response to oxygen levels (GO:0070482), ovarian steroidogenesis (ko04913), and ovulation (GO:0030728) (Figure 5, Table S9), implying that these highly expressed genes could contribute to follicle maturation and ovulatory process. For example, *RGS2* was involved in the ovulation events [59], and *STAR* was the highest expressed DEG at the ZT20 stage (Figure 6c), which is related to steroidogenesis and encodes a steroidogenic acute regulatory protein that is instrumental in steroid hormone synthesis and ovulation during follicle development [67,68,69].

Furthermore, gene expression correlation coefficients were calculated to directly predict the functional correlation of lncRNAs across three stages of the ovulatory cycle (Figure 6). The highly abundant lncRNA *G13560* had similar expression patterns to the steroid-related genes in the RNA-seq data over the three stages, which showed the highest expression at the ZT20 stage (Figure 6b). This implied that these genes involved in steroids regulation associated with changes in serum LH levels. Due to the lack of lncRNA annotation, especially lncRNA in farm animals like chickens, the co-expressed targets of lncRNA could be to predict the lncRNA functions. The abundance of *G13560* and *STAR* with a high correlation coefficient over the ovulatory cycle were highly expressed in the ZT20 stage. Then, the cultured cells were treated with LH, and both the co-expressed genes were also upregulated in vitro (Figure 6d). Thus, we propose that *G13560* and *STAR* may co-regulate the functions activated by LH surge-induced cascade in granulosa cells. All of the above findings suggested that the biological functions of the DEGs and DELs regulate follicle development in chickens during the ovulatory cycle.

## 5. Conclusions

In the present study, the chicken preovulatory follicle (F1) underwent the ZT0, ZT12, and ZT20 stages during the ovulatory cycle. The study provides a comprehensive analysis of lncRNAs and mRNAs in chicken granulosa cells. Thousands of lncRNAs were annotated, and the most differentially abundant genes were detected in the ZT20-ZT12 group, and functional features of the lncRNAs and mRNAs at each stage were revealed, which was also associated with the changes in serum LH level. Furthermore, the expression of lncRNA *G13560* and its co-expressed mRNA were verified in cultured granulosa cells with LH treatment. The comprehensive data generated in this study provides the foundation for future investigations to improve the reproductive performance of chickens and explore the mechanisms responsible for female ovarian diseases.

## Figures and Tables

**Table 1:** Data summary of RNA-seq in granulosa cell of chickens.

Samples	Raw Reads	Raw Base(G) ^1^	Q20(%) ^2^	Q30(%) ^3^	GC Content(%) ^4^
ZT0_1	54,270,570	16.28	97.56	93.34	48.82
ZT0_2	56,376,040	16.91	97.34	92.95	48.92
ZT0_3	60,354,655	18.11	97.13	92.45	47.84
ZT12_1	67,301,276	20.19	97.35	92.91	51.37
ZT12_2	47,927,165	14.38	97.44	93.17	52.69
ZT12_3	61,112,368	18.33	97.44	93.17	55.09
ZT20_1	58,567,244	17.57	97.59	93.41	48.12
ZT20_2	53,311,100	15.99	97.53	93.27	47.93
ZT20_3	52,926,730	15.88	97.17	92.61	54.05

^1^ G, Giga base; ^2^ Q20, a quality score of 20 represents an error rate of 1 in 100, with a corresponding call accuracy of 99%; ^3^ Q30, a quality score of 30 represents an error rate of 1 in 1000, with a corresponding call accuracy of 99.9%; ^4^ GC, the proportion of guanine (G) and cytosine (C) bases in the four DNA bases, also including adenine and uracil in RNA. ZT0 (9:00 a.m.), represents the early stage of the ovulatory cycle; ZT12 (9:00 p.m.), represents the middle stage of the ovulatory cycle; ZT20 (5:00 a.m.), represents the LH surge stage.

## Data Availability

The raw reads generated for this study were deposited in the National Center for Biotechnology Information (NCBI) Gene Expression Omnibus (GEO) with accession number GSE152268 (https://www.ncbi.nlm.nih.gov/geo/query/acc.cgi?acc=GSE152268, accessed on 28 April 2021). The remaining data that support the findings of this study are available from the corresponding author upon reasonable request.

## Supplementary Materials

The following are available online at https://www.mdpi.com/article/10.3390/ani11061533/s1, Additional file 1: the custom scripts and options of bioinformatics tools in this study. Additional file 2: Genomic characteristics of lncRNAs and mRNAs; Additional file 3: The sequences of identified lncRNAs; Additional file 4: The number of DE-lncRNAs and DEGs in different clusters produced by STEM; Table S1: Primer sequences of selected transcripts for qPCR; Table S2: The information of all the identified transcripts in chicken follicle; Table S3: Differentially expressed mRNAs in pairwise stages; Table S4: Functional categories of differentially expressed mRNAs in pairwise stages; Table S5: Differentially expressed lncRNAs in pairwise stages; Table S6: Functional categories of DEGs co-localized with DE-lncRNAs; Table S7: Lists of mRNAs in different cluster profiles of STEM; Table S8: Lists of lncRNAs in different cluster profiles of STEM; Table S9: Functional categories of differentially expressed mRNAs in cluster 10 of STEM.

## Abbreviations

lncRNA	Long Non-coding RNA
LH	Luteinizing hormone
ZT	Zeitgeber time
ZT0	The early stage of the ovulatory cycle at 9:00 a.m.
ZT12	The middle stage of the ovulatory cycle at 9:00 p.m.
ZT20	The LH surge stage at 5:00 a.m.
LH	Luteinizing Hormone
TPM	Transcripts Per Kilobase Million
STEM	Short time-series expression miner
DEGs	Differentially expressed genes
DELs	Differentially expressed lncRNAs
DMEM	Dulbecco’s modified Eagle’s medium
DXM	Dexamethasone
qPCR	Quantitative real-time PCR
H&E	Hematoxylin and Eosin
t-SNE	t-Distributed stochastic neighbor embedding
GO	Gene Ontology
PDGFA	Platelet-Derived Growth Factor Subunit A
FASN	Fatty Acid Synthase
RGS16	Regulator of G protein signaling 16
EGFR	Epidermal Growth Factor Receptor
RGS2	Regulator of G Protein Signaling 2
STAR	Steroidogenic Acute Regulatory Protein
KEGG	Kyoto Encyclopedia of Genes and Genomes
